# Comprehensive profiling of antibiotic resistance, virulence genes, and mobile genetic elements in the gut microbiome of Tibetan antelopes

**DOI:** 10.1128/msystems.01443-25

**Published:** 2025-12-23

**Authors:** Jian Liu, Hong-Bo Ni, Ming-Yuan Yu, Si-Yuan Qin, Hany M. Elsheikha, Peng Peng, Li Guo, Lin-Hong Xie, Hong-Rui Liang, Cong-Cong Lei, Yu Xu, Yan Tang, Hai-Long Yu, Ya Qin, Jing Liu, Hong-Chao Sun, Xiao-Xuan Zhang, Bin Qiu

**Affiliations:** 1 College of Veterinary Medicine, Qingdao Agricultural University 98431, Qingdao, Shandong Province, PR China; 2 Center of Prevention and Control Biological Disaster, State Forestry and Grassland Administration 842433 , Shenyang, Liaoning, PR China; 3 Faculty of Medicine and Health Sciences, School of Veterinary Medicine and Science, University of Nottingham, Sutton Bonington Campus 6123, Loughborough, United Kingdom; 4 Animal Science and Technology College, Jilin Agricultural Science and Technology University 381876, Jilin, PR China; 5 College of Pharmacy, Guizhou University of Traditional Chinese Medicine 326770 , Guiyang, Guizhou, PR China; 6 College of Veterinary Medicine, Jilin Agricultural University 85112, Changchun, Jilin, PR China; 7 College of Life Sciences, Changchun Sci-Tech University 381875, Shuangyang, Jilin, PR China; 8 Institute of Animal Husbandry and Veterinary Medicine, Zhejiang Academy of Agricultural Science 823338 , Hangzhou, Zhejiang, PR China; 9 College of Chemistry and Pharmaceutical Sciences, Qingdao Agricultural University 98431, Qingdao, Shandong, PR China; Peking University, Beijing, China

**Keywords:** Tibetan antelope, Plateau region, Antibiotic resistance genes, Virulence factor genes, Mobile genetic elements

## Abstract

Tibetan antelopes, native to high-altitude plateau regions, play an important role in the local ecosystem. Their gut harbors antimicrobial-resistant microbes, including potential pathogens. To explore this, we analyzed 33,925 metagenome-assembled genomes (MAGs), including 7,318 from 68 Tibetan antelopes sequenced in our laboratory. We first profiled the composition of antibiotic resistance genes (ARGs) and then examined their associations with virulence factor genes (VFGs). In total, 2,968 ARGs were identified, conferring resistance to 23 antibiotic classes, with elfamycin resistance being most prevalent. Two ARGs were located on phage-derived sequences, though their phage taxonomy could not be resolved. ARGs were significantly correlated with VFGs, particularly genes linked to adherence and effector delivery systems. Given potential dissemination risks, we further assessed associations between ARGs and mobile genetic elements (MGEs), finding that insertion elements accounted for the largest number of ARG–MGE links. Comparative analysis with other plateau animals and humans revealed seven ARGs uniquely present in Tibetan antelopes. In summary, this study provides the first comprehensive overview of ARG composition in Tibetan antelope gut microbiomes, establishing a baseline for future hypothesis-driven studies and antimicrobial resistance surveillance in wildlife.

**IMPORTANCE:**

Investigating the drug resistance of Tibetan antelope (*Pantholops hodgsonii*) gut microbiota serves as a critical biological indicator for assessing the impact of human activities (particularly antibiotic contamination) on the fragile ecosystem of the Qinghai-Tibet Plateau. This study untangles the invasion of antibiotic resistance genes (ARGs) into remote conservation areas, suggesting that Tibetan antelopes may act as potential vectors for ARG dissemination across plateau environments. Such findings not only highlight threats to wildlife health but also provide an ecological warning regarding the pervasive environmental risks posed by the global antimicrobial resistance crisis in natural ecosystems.

## INTRODUCTION

Antibiotics are among the most influential medical discoveries of the 20th century, having saved millions of lives by combating bacterial infections (1). However, the overuse and misuse of antibiotics have led to a steady rise in antibiotic resistance genes (ARGs), posing a serious global health threat. Since the early 21st century, the growing prevalence of antibiotic resistance has raised concerns about the diminishing effectiveness of current treatments, leaving humanity vulnerable to untreatable infections (2). The rapid spread of ARGs has become a critical public health crisis, with significant implications for both human and environmental health (3).

ARGs are naturally present in diverse ecosystems, where they serve as a defense mechanism against antibiotics produced by other microorganisms (4–6). The origins of resistance genes can be traced back millions—even billions—of years, yet their widespread dissemination has accelerated due to anthropogenic influences (6, 7). Through horizontal gene transfer, ARGs can spread across microbial communities, creating reservoirs of resistant bacteria (5). While antibiotic resistance has historically been linked to agricultural and healthcare settings, recent studies indicate that ARGs are now prevalent even in remote natural environments. This suggests that indirect human activities and environmental factors may contribute to their dissemination (8).

Beyond human and environmental influences, animals also play a crucial role in the spread of ARGs. Wildlife, particularly species in isolated or extreme habitats, may serve as unexpected reservoirs of antibiotic resistance, facilitating the movement of ARGs beyond human-dominated landscapes (9). Understanding how natural environments contribute to or mitigate the spread of resistance genes is essential for addressing the broader implications of antibiotic resistance.

The Qinghai-Tibet Plateau (QTP), often referred to as the “Roof of the World,” is one of Earth’s most extreme and ecologically unique regions, averaging over 4,000 m in altitude. This vast, high-altitude plateau spans western China, bordering India in the west and the Kunlun, Arjin, and Qilian Mountains in the northeast and northwest (10). The plateau’s harsh conditions—low oxygen levels, intense ultraviolet radiation, and extreme cold—have led to the evolution of highly specialized flora and fauna adapted to survive in this environment (11). Importantly, these climatic stressors can also shape microbial communities and their resistomes; for instance, ultraviolet (UV) radiation is a critical factor affecting bacterial survival, as it induces oxidative stress and DNA damage, which may facilitate the acquisition and dissemination of ARGs (12). Moreover, recent studies have shown that environmental factors, such as altitude, water temperature, light intensity, and dissolved oxygen, influence resistome similarity, which tends to decrease with increasing altitude. Despite its relative isolation, the QTP is increasingly exposed to human activities, such as livestock grazing, tourism, and antibiotic use in agriculture, which may introduce antibiotic residues and resistant bacteria into the ecosystem. These unique ecological conditions provide an exceptional opportunity to study the distribution and persistence of ARGs in a natural setting, as well as their potential transmission within and between wildlife species.

The Tibetan antelope (*Pantholops hodgsonii*), an iconic species endemic to the QTP, is of particular interest in this study due to its ecological significance and conservation status. Listed in the Convention on International Trade in Endangered Species since 1981, the Tibetan antelope has experienced population recovery in recent years and is now classified on the IUCN Red List of Endangered Species (13, 14). Adapted to the extreme conditions of the plateau, this species helps maintain the structure and stability of the local ecosystem through its feeding habits and interactions with natural predators. Given its exposure to diverse microbial communities, the Tibetan antelope may act as a reservoir or vector for ARGs, contributing to their distribution across the plateau. However, research on ARG dynamics in Tibetan antelopes remains limited, particularly in comparison to other plateau-dwelling species.

This study aims to explore the role of Tibetan antelopes in the transmission and distribution of ARGs within the Qinghai-Tibet Plateau ecosystem. By analyzing bacterial diversity and ARG abundance in Tibetan antelopes and comparing these findings with data from five other plateau species, we seek to identify unique patterns of ARG transmission and assess the ecological risks associated with ARG-host relationships. Mobile genetic elements (MGEs), which mediate the movement of DNA within and between cells (e.g., from chromosomes to plasmids or across plasmids), play an important role in the capture, accumulation, and dissemination of ARGs. In parallel, ARGs and virulence factor genes (VFGs) are considered key microbial indicators: ARGs enable bacteria to withstand antibiotic pressure, while VFGs allow pathogens to invade hosts and cause disease. Therefore, we examine the interactions between ARGs, MGEs, and VFGs to understand their potential role in the dissemination of antibiotic resistance in this extreme environment. Additionally, viruses serving as important reservoirs of ARGs (15, 16) see their horizontal gene transfer generally mediated by transduction via phages—viruses that infect bacteria (17). Therefore, predicting viral sequences and detecting ARGs were necessary to investigate the drug resistance profile in Tibetan antelopes.

The findings from this study will provide valuable insights into the ecological roles of wildlife in ARG spread and contribute to a broader understanding of antibiotic resistance in remote and extreme environments. This research not only establishes foundational knowledge about ARGs within Tibetan antelopes but also offers a new perspective on the mechanisms driving antibiotic resistance in high-altitude ecosystems. The results may have broader implications for global studies on ARG dissemination and wildlife-associated resistomes.

## MATERIALS AND METHODS

### Tibetan antelope sample collection

Freshly excreted feces of Tibetan antelopes were located using a telescope. Subsequently, fecal samples were carefully collected using sterile tweezers and transferred into labeled sterile centrifuge tubes, with each sample stored in a separate tube. A total of 68 fecal samples were obtained from Tibetan antelopes across Tibet, Qinghai, and Xinjiang (Table S2). All samples were stored at −80°C until further processing.

### Metagenomic sequencing and data analysis

Total DNA was extracted from the 68 fecal samples collected in this study using the OMEGA Mag-Bind Soil DNA Kit (M5635-02, Omega Bio-Tek, Norcross, GA, USA) following the manufacturer’s instructions. DNA concentration and purity were measured using a Qubit 4 Fluorometer and verified by 1% agarose gel electrophoresis. Samples meeting quality control standards were adjusted to a final concentration of 10 nM for library preparation. Metagenomic sequencing was performed on the Illumina HiSeq platform using paired-end (2 × 150 bp) sequencing, generating approximately 10 Gb of raw data per sample.

Fastp (v0.23.0) was used to filter for high-quality reads, which were then processed with Bowtie2 (v2.5.0) to remove host genomic (PHO1) DNA contamination. Contigs were assembled using MEGAHIT (v1.2.9) (18), and sequencing depth files were generated with BWA (v0.7.17-r1198) (19), SAMtools (v1.18) (20), and the script jgi_summarize_BAM_contig_depths. Binning was conducted with MetaBAT2 (v2.15) (21) using the parameters -m 2000 -s 200000 --seed 2023. The completeness and contamination of each bin were assessed using CheckM2 (v1.0.1) (22), retaining bins with completeness ≥50% and contamination ≤10% (23).

This study obtained 7,386 metagenome-assembled genomes (MAGs), and 26,607 public Tibetan antelope MAGs were dereplicated at 99% average nucleotide identity (ANI) was performed using dRep (v3.4.3) (24) with the parameters -pa 0.9 -sa 0.99. Taxonomic classification of the dereplicated MAGs (99% identity clusters) was performed using Genome Taxonomy Database (GTDB)-Tk with default parameters (25), referencing the GTDB to assign taxonomic ranks from domain to species. Phylogenetic trees were constructed at the amino acid level using PhyloPhlAn (v.1.0).

### Public data collection and pre-processing of sequencing reads

In addition to the 68 fecal samples collected from Tibetan antelopes in this study, 255 Tibetan antelope samples and 26,607 MAGs were retrieved from the China National GeneBank (CNGB) Sequence Archive under project accession number CNP0001390 (26). Fecal metagenomic data from other herbivores were obtained from the CNGB database. These included samples from *P. hodgsonii* and five other large herbivore species: yak (*Bos grunniens*, 28,125 MAGs), Tibetan wild ass (*Equus kiang*, 6,684 MAGs), Tibetan sheep (*Ovis aries*, 39,378 MAGs), Tibetan cattle (*Bos taurus*, 10,630 MAGs), and Tibetan horse (*Equus caballus*, 8,144 MAGs).

Furthermore, 60,664 high- and medium-quality MAGs of human (10.1038/s41586-019-1058-x) were also obtained. Additionally, 32 fecal metagenomic data sets from Tibetan human individuals were obtained from NCBI, consisting of 17 samples from PRJEB53209 (27) and 15 from PRJNA543906 (28). All Tibetan humans' fecal metagenomic data sets were processed using the same standardized data analysis pipeline as applied to the 68 fecal samples from Tibetan antelopes. For the Tibetan human samples, host-derived reads were removed by mapping against the GRCh38 reference genome using Bowtie2 (29) (v2.4.1) with default parameters.

### Identification and processing of viral sequences

To identify viruses potentially involved in the dissemination of antimicrobial resistance, established methods were used to screen contigs >5,000 bp from 2,149 MAGs carrying ARGs (30, 31). Initially, CheckV (v1.0.1) was used to assess the ratio of viral to host genes (32). Contigs containing more than 10 host genes or where host genes outnumbered viral genes by more than fivefold were excluded. Proviral fragments were also identified using CheckV. Next, multiple viral detection strategies were employed, including (i) viral gene enrichment as determined by CheckV, (ii) identification by DeepVirFinder (v1.0.19) (33) with a score greater than 0.90 and *P* value less than 0.01, and (iii) viral classification by VIBRANT (v1.2.1) (34) using default parameters. Contigs meeting any of these criteria were retained as putative viral sequences. To remove potential bacterial contamination, BUSCOs (35) were used in combination with hmmsearch to detect bacterial single-copy orthologs within the viral candidates. The BUSCO ratio (calculated as the number of BUSCOs divided by total gene count) was computed, and sequences with a ratio of 5% or higher were excluded. The remaining sequences underwent quality assessment with CheckV, and only viral genomes with medium or higher completeness were retained for downstream analyses.

### Functional annotation

Gene prediction for all MAGs obtained in this study was performed using Prodigal (10.1186/1471-2105-11-119) (v2.6.1). ARGs were identified by aligning protein sequences against the Comprehensive Antibiotic Resistance Database (CARD v3.2.7) (36) using DIAMOND (v2.1.8.162) (25), with a threshold of >80% sequence identity and >80% query coverage (e-value = 1e-5) (37). Multidrug resistance genes were defined as those conferring resistance to at least two antibiotic classes, while multitype mechanism genes conferred resistance via at least two distinct mechanisms. MGEs were detected by aligning gene sequences to the MGE Database (38) using BLASTN (v2.13.0) (39) with the parameters -evalue 1e-5 -perc_identity 80 -qcov_hsp_perc 80. VFGs were identified by aligning sequences to the Virulence Factor Database (VFDB) (40) using DIAMOND (v2.1.8.162), with a sequence identity threshold of >80% and query coverage >80%.

To generate taxonomic profiles for Tibetan antelope and human-derived ARGs, clean reads from each sample were mapped to reference ARG sequences using Bowtie2 (v2.4.1) with the parameters --end-to-end --fast --no-unal. Total mapped reads across all samples were normalized to the same sequencing depth. The read counts were normalized to transcripts per kilobase million.

### Statistical analyses and visualization

The resulting phylogenetic trees were annotated and visualized using iTOL (41). Procrustes association analysis was conducted on the profiles of ARGs, MGEs, and VFGs, utilizing the “procrustes” function from the “vegan” package. Taxonomic assignments of genomes containing ARGs were identified, and Sankey plots were generated using the “ggsankey” package (v0.0.9). The Richness index was calculated from the relative abundance of functional genes. The correlation between ARGs, VFGs, and MGEs was assessed using the cor.test function in R, with Spearman’s rank correlation method. Gene arrow maps were constructed using the “gggenes” (v0.4.1) package. GCView services (https://proksee.ca/) were used to visualize the genome and to mark ARGs, MGEs, and VFGs. Network visualization of these correlations was conducted using the igraph v2.14.0 package. All other visualizations were produced with the ggplot2 package version 3.3.6. Statistical analyses were performed using R version 4.4.1.

## RESULTS

### Comprehensive gut genome of the Tibetan antelope

**Fig 1:**
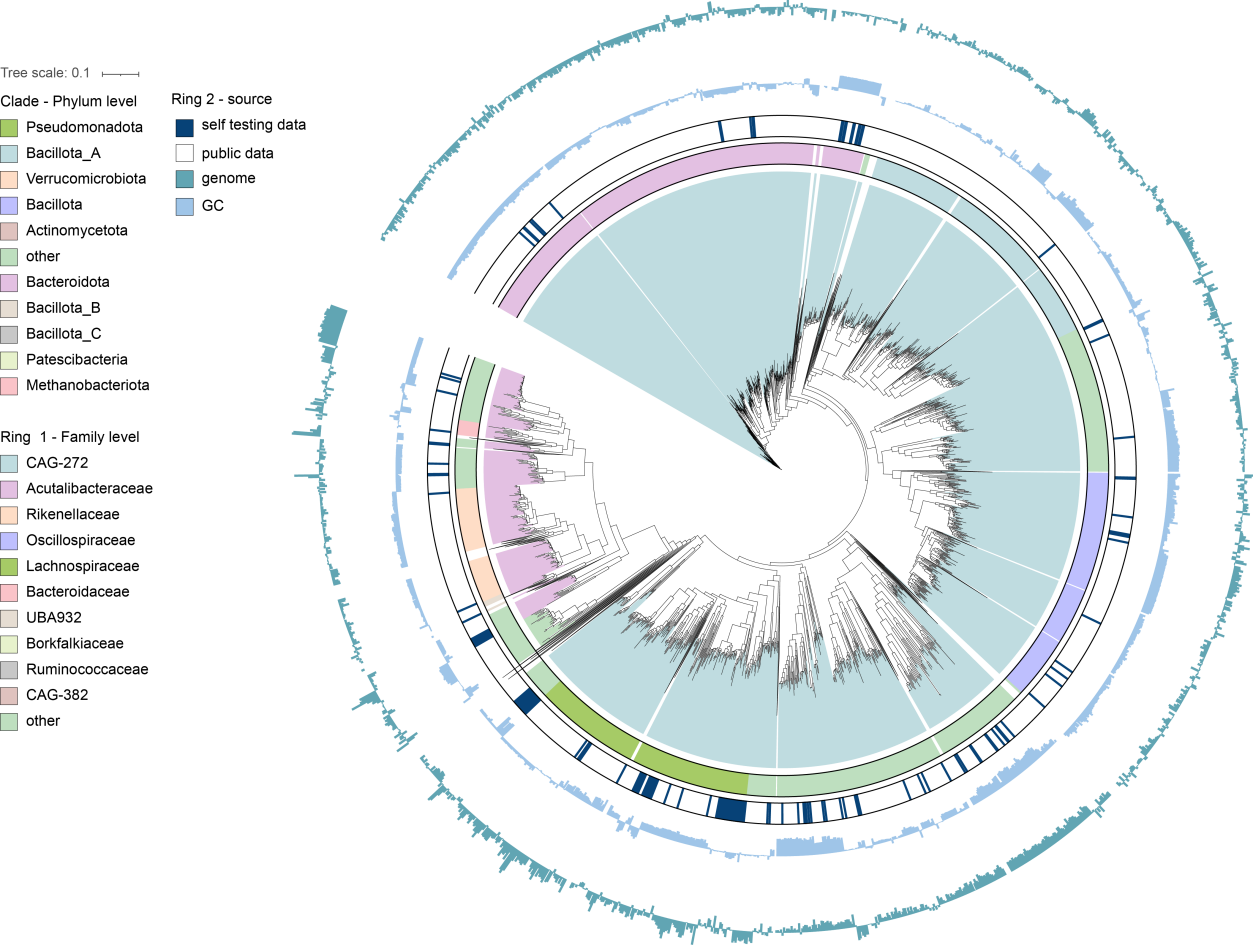
MAGs of Tibetan antelope. The phylogenetic relationship among the 13,600 bacterial genomes. The color coding of each clade corresponds to the phylum-level classification of the genomes. The first outer ring denotes family-level classification. The second outer ring denotes the genome source, distinguishing between self data and public data. The third and fourth rings are bar charts representing the GC content and the genome size of each genome, respectively.

A total of 33,925 MAGs from Tibetan antelope gut microbiota were obtained from public databases (see Materials and Methods). After quality control using CheckM2, 28,752 medium- to high-quality MAGs (completeness ≥50% and contamination ≤10%) were retained. These MAGs were clustered at 99% ANI, resulting in 13,600 strain-level genomes (Fig. 1). The average completeness of these genomes was 82.50%, with a mean contamination of 1.95% (Fig. S1B). Genome sizes ranged from 0.38 to 6.50 Mbp (average 1.89 Mbp/genome), while GC content varied between 23.93% and 73.57% (average 45.82%) (Fig. S1C).

Taxonomic classification using GTDB-Tk assigned these genomes to 2 domains, 21 phyla, 28 classes, 76 orders, 164 families, and 674 genera. At the domain level, 14,234 genomes were classified as bacteria, while 335 genomes belonged to archaea (Table S3). The dominant phylum was *Bacteroidota_A* (7,963 genomes, 58.55%), followed by *Bacteroidota* (3,488 genomes, 25.65%). At the genus level, *Alistipes* had the highest representation (1,208 genomes, 8.88%), followed by *Faecousia*, *Cryptobacteroides*, and *Scatosoma* (Fig. S1D). Notably, 343 genomes (2.52%) remained unclassified at the genus level, indicating the presence of potentially novel microbial taxa in the Tibetan antelope gut microbiome. Among the MAGs analyzed in this study, 12,623 were obtained from public databases, while 977 were generated from our own sequencing. To enable comparison, the two datasets were analyzed separately. The results showed that the proportion of archaea was relatively higher in our sequencing data (7.4%) than in the public data (2.1%). At the phylum level, Actinomycetota was markedly more abundant in the self-sequenced data set, accounting for 8.1%, whereas its proportion in the public data set was below 1%. At the species level, both data sets contained a large proportion of unclassified taxa, which may be related to the limited research available on microbiota associated with high-altitude animals.

### Composition of ARGs in Tibetan antelope

**Fig 2:**
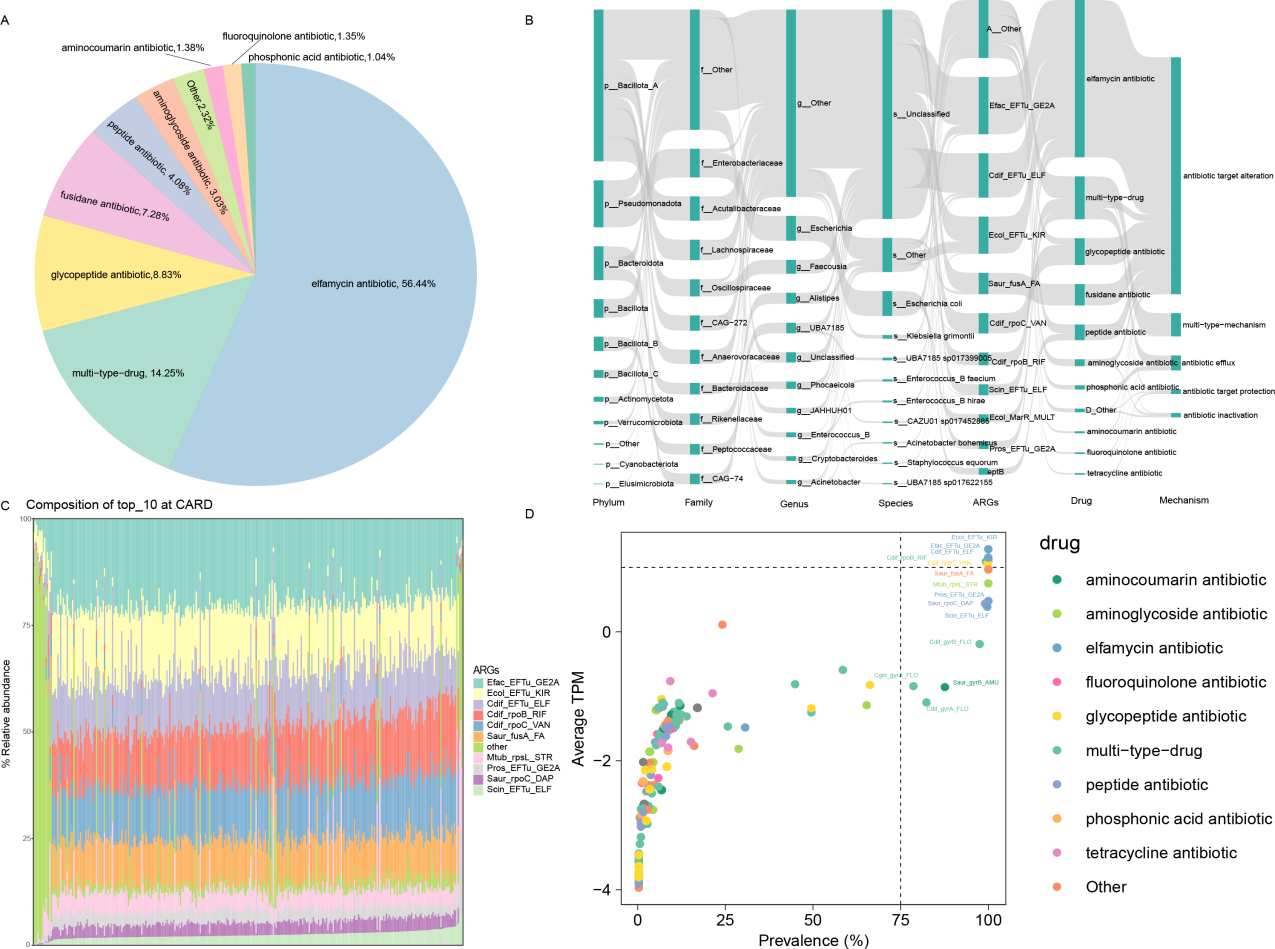
Composition of ARGs in Tibetan antelope. (**A**) Proportion of resistance to different drug classes. (**B**) Host classification of ARGs, including their resistance mechanisms. (**C**) Composition of the top 10 ARGs. (**D**) Prevalence and abundance distribution of all ARGs in the 323 tested samples. The abundance of each ARG is represented as the mean value across all samples. Dots of different colors represent distribution patterns based on abundance levels and frequencies.

We compared 13,600 genomes to the CARD and identified 2,968 ARGs. These genes confer resistance to 23 different antibiotics (Table S4). The ARGs were classified into five resistance mechanisms, excluding multidrug resistance. Antibiotic target alteration was the most prevalent mechanism, present in 2,478 genomes (83.49% of all resistance mechanisms). The least common mechanism was reduced permeability to antibiotics, found in only 12 genomes (0.40%). Multidrug resistance ranked second, with 212 genomes (7.14%) exhibiting this mechanism (Table S4). When analyzed separately, the self-sequenced data set yielded 596 ARGs, accounting for 19.2% of the total, while 2,399 ARGs were identified in the public data set. Regarding resistance types, ARGs in the public data set conferred resistance to 19 antibiotic classes, whereas those in the self-sequenced data set conferred resistance to 23 classes, four more than in the public data set. Nevertheless, in both data sets, the most prevalent resistance was against elfamycin antibiotics. We categorized the types of antibiotics these resistance genes target. The most prevalent resistance was against elfamycin antibiotics, comprising 56.44% of the total, followed by multi-type drug resistance (14.25%), glycopeptide antibiotics (8.83%), fusidane antibiotics (7.28%), and peptide antibiotics (4.08%). The least common resistance was to phosphonic acid antibiotics, found in 31 genomes (1.04%) (Fig. 2A).

Host prediction analysis at the phylum level revealed that *Bacillota_A* contained the highest number of ARGs, followed by *Pseudomonadota*, *Bacteroidota*, *Bacillota*, and *Bacillota_B*. At the species level, a significant proportion of ARGs could not be clearly classified. Among those that were classified (excluding unresolvable species), *Escherichia coli* harbored the largest number of ARGs, particularly resistant to peptide antibiotics. In addition to this, *E. coli* exhibited resistance to multiple other antibiotics as well (Fig. 2B).

To further investigate the composition of ARGs, we examined the distribution of the top 10 ARGs in each Tibetan antelope sample (Fig. 2C). In over 75% of the samples, the proportion of *Efac_EFTu_GE2A* exceeded 12.5%. This was followed by *Efac_EFTu_KIR* and *Cdif_EF_ELF*, with the combined proportion of these three ARGs surpassing 50% in nearly half of the samples.

The top 10 ARGs confer resistance to five types of antibiotics: elfamycin, peptide, rifamycin, glycopeptide, and aminoglycoside antibiotics. The primary resistance was observed toward elfamycin antibiotics, with genes, such as *Ecol_EFTu_KIR*, *Cdif_EFTu_ELF*, *Pros_EFTu_GE2A*, and *Scin_EFTu_ELF,* contributing to this resistance. Additionally, *Cdif_rpoB_RIF* provides resistance to both peptide antibiotics and rifamycin antibiotics. Except for *Cdif_rpoB_RIF*, all other ARGs primarily exhibit resistance through antibiotic target alteration. *Cdif_rpoB_RIF*, however, involves both antibiotic target alteration and a secondary mechanism of antibiotic target replacement. The prevalence of these ARGs was detected in over 75% of the samples (Fig. 2D).

Additionally, some MAGs contained phage sequences. Viral sequence prediction was performed on the MAGs carrying ARGs, identifying a total of 7,802 viral sequences. Following clustering and annotation, resulting in 2,936 non-redundant sequences, the majority of these sequences were found to belong to the *Siphoviridae* family. These clustered viral sequences were then aligned against the CARD database, revealing two viral sequences (taxonomic family is unknown) harboring ARGs. These ARGs conferred resistance to fusidane antibiotics and lincosamide antibiotics, respectively (Table S4).

### ARGs diversity across Tibetan antelope, human, and other Tibetan mammals

**Fig 3:**
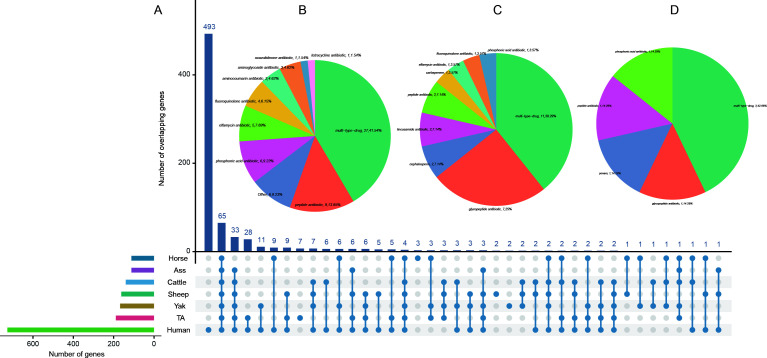
MAGs and ARGs analysis of other Tibetan mammals and humans. (**A**) Comparison of ARGs between Tibetan antelopes and other species, with the vertical axis representing common ARGs across species. (**B**) Shared ARGs between plateau-dwelling animals and human populations. (**C**) Shared ARGs between Tibetan antelope and human populations. (**D**) ARG types are exclusively present in the gut microbiota of Tibetan antelopes.

The methods for processing the MAGs of other plateau animals, including Tibetan wild asses, yaks, Tibetan wild horses, and Tibetan wild cattle, were consistent with those used for Tibetan antelope MAGs. Among the plateau animals, Tibetan sheep had the highest number of MAGs, with 39,378 MAGs collected, and the largest number of ARGs mapped to them (5,961 ARGs). This was followed by Tibetan yak (4,935 ARGs) and Tibetan wild cattle (2,009 AGRs) (Table S10 through S13). Further analysis of ARGs in these plateau-dwelling animals and human populations revealed that, excluding those categorized as “others,” the top three most prevalent ARGs were primarily associated with multi-type-drug resistance, peptide antibiotics, and phosphonic acid antibiotics (Fig. 3A and B).

Comparative analysis of shared ARGs between Tibetan antelopes and human populations identified 28 distinct ARG types. Among these, 11 ARGs conferred resistance to multiple drug classes, while 7 ARGs specifically targeted glycopeptide antibiotics (Fig. 3C). Notably, we identified 7 ARGs exclusively present in Tibetan antelopes, with no counterparts detected in other plateau-dwelling species or Tibetan populations. Particularly significant were three multidrug-resistant ARGs in Tibetan antelopes demonstrating simultaneous resistance to carbapenems, cephalosporins, and penams. Subsequently, BLAST analysis of the shared ARGs between Tibetan antelopes and humans via NCBI identified a vancomycin-resistant Enterococcus gallinarum strain and a tigecycline-resistant *Bacteroides clarus* strain (Fig. S2A and B; Table S9).

### MGEs related to ARGs in the gut of Tibetan antelope

**Fig 4:**
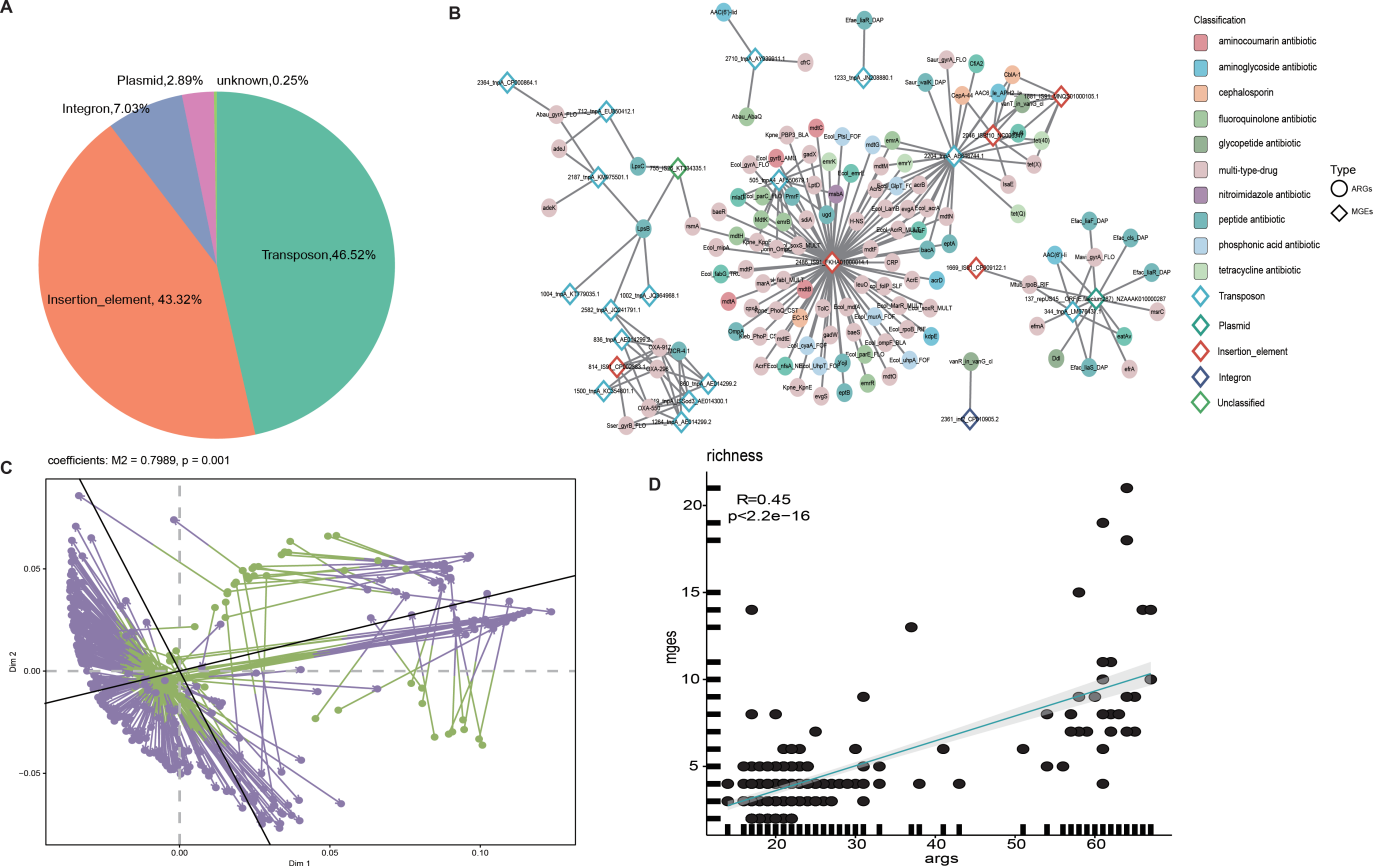
MGEs related to ARGs. (**A**) Abundance of different types of MGEs across samples. (**B**) Correlation network between MGEs and ARGs. ARGs are represented by circles, and MGEs by rhombuses. (**C**) Procrustes association analysis: correlations between ARGs and MGEs in multidimensional space. (**D**) Spearman’s correlation analysis of the richness index between MGEs and ARGs.

MGEs play an important role in the horizontal transfer of ARGs, both within bacterial populations and across different species. Understanding their distribution patterns and their relationship with ARGs is essential for comprehending the mechanisms of antibiotic resistance spread. In this study, a total of 132 MGEs were identified from 13,600 MAGs by aligning protein sequences with the MGE database. These MGEs were classified into five categories: insertion elements, transposons, integrons, plasmids, and unclassified MGEs (Table S6). Transposons, characterized by transposase genes, were the most abundant MGE type in the Tibetan antelope gut microbiome, comprising 46.52% of the total MGE abundance. This was followed by Insertion_element (43.32%), integrons (7.03%), Plasmids (2.89%), and unknown elements (0.25%) (Fig. 4A).

We then conducted a correlation analysis between the MGEs and ARGs, focusing on relationships with a corr >0.6 and a *P* value <0.05 (Fig. 4B). The analysis showed that Insertion_elements were associated with a higher number of ARGs compared to other MGE types. Notably, ARGs strongly correlated with Insertion_elements mainly include multi-type-drug and peptide antibiotic resistance genes. Furthermore, procrustes analysis revealed a strong association between the cecal mobilome and antibiotic resistance (PROTEST, M² = 0.7989, *P* = 0.001) (Fig. 4C). This was supported by a significant positive correlation between the MGE and ARG profiles, as measured by the Richness index (Spearman’s correlation: R = 0.45, *P* < 2.2^e-16^) (Fig. 4D). Additionally, ARGs located within 5 kilobases (kb) of an MGE were classified as potentially mobile. Only one Escherichia coli strain (X23.bin.1) was identified carrying two ARG–MGE configurations: Ecol_emrE (conferring resistance to macrolide antibiotics) paired with ISSfl3 and AcrF (associated with multi-type-drug resistance) linked to IS91. Notably, the AcrF gene demonstrated resistance to cephalosporins, cephamycins, fluoroquinolone antibiotics, and penams (Fig. S2C). While ARG–MGE associations are generally common, the presence of multiple such configurations in *E. coli*, an important opportunistic pathogen, highlights its potential role as a reservoir for mobilizable resistance genes.

### Composition of VFGs and their relationship with ARGs in Tibetan antelope

**Fig 5:**
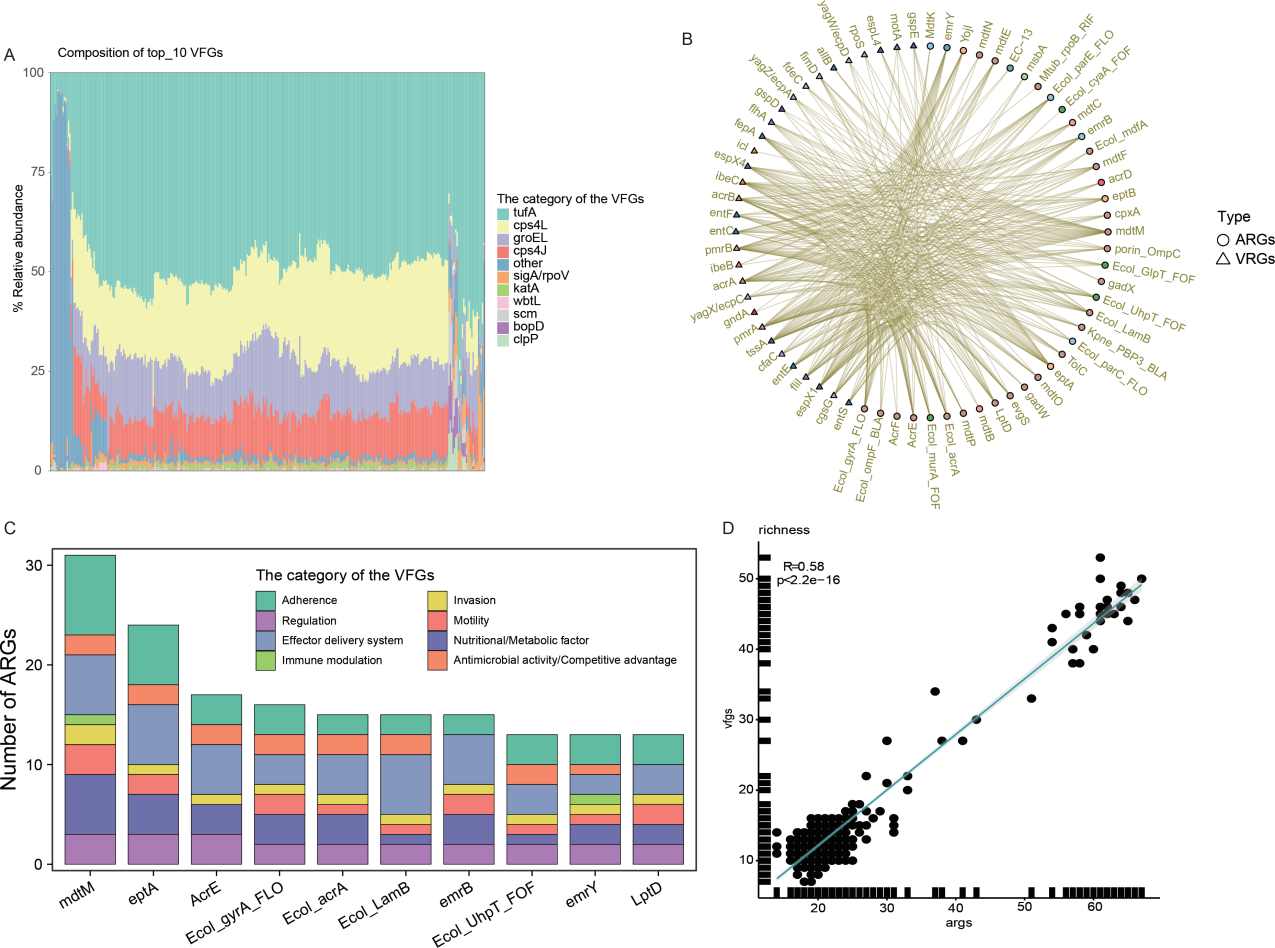
Composition of VFGs and correlation between ARGs and VFGs. (**A**) Abundance of VFs in Tibetan antelope samples. (**B**) Correlation between ARGs and VFGs, with corr >0.8 and *P* value <0.05. (**C**) Number of correlations for the top 10 ARGs, with bars representing the number of each category of VFGs associated with the ARGs. (**D**) Spearman’s correlation analysis between the richness index of VFGs and ARGs.

To further analyze the composition of VFGs in the gut metagenome of Tibetan antelopes, we identified 4,729 VFGs by comparing the metagenomes with the VFDB. Among these, the most abundant VFG was *tufA*, which accounted for 48.18% of the VFGs (Table S8). This gene was present in nearly all Tibetan antelope samples, with its relative abundance exceeding 25% in most cases (Fig. 5A). The second most prevalent VFG was *cps4L*, accounting for 19.86%, followed by *groEL* at 12.06%, *cps4J* at 9.85%, and *sigA/rpoV* at 1.43%. Based on functional classifications, the two virulence factor genes, *tufA* and *groEL,* are primarily associated with adherence functions, while *cps4L* and *cps4J* are linked to immune modulation. sigA/rpoV, on the other hand, is involved in regulatory functions (Table S8).

To examine the relationship between ARGs and VFGs, we performed a correlation analysis using Spearman’s method. We focused on relationships with a correlation coefficient (corr) >0.8 and a *P* value <0.05 (Fig. 5B). We found that all the top 10 ARGs showed significant associations with VFGs related to adherence functions, with the highest number of such associations (Fig. 5C). The second most common association was with virulence genes related to the effector delivery system. Additionally, several of these ARGs confer resistance to multiple drugs, such as *mdtM*, *Ecol_acrA*, *Ecol_gyrA_FLO*, *Ecol_LamB*, and LptD (Table S4). Our study revealed a significant positive correlation between the profiles of VFGs and ARGs based on the Richness index (Spearman’s correlation: R = 0.58, *P* < 2.2^e-16^) (Fig. 5D). Applying the same analytical approach to examine associations between VFGs and MGEs, five distinct MGE-VFG combinations were identified across three MAGs. These combinations correspond to three categories of MGEs and VFGs, including tnpA (Transposon), tnpA4 (Transposon) paired with TTSS secreted effectors (Effector delivery system), and ISSfl3 (Insertion_element) paired with StcE (Exoenzyme) (Fig. S2F). Notably, both strains harbored over 65 ARGs each, a characteristic that may have significant public health implications.

## DISCUSSION

A total of 13,600 MAGs were analyzed in this study, including 977 from our own sequencing of Tibetan antelopes and 12,623 from public data sets. Although both originated from the Qinghai-Tibet Plateau, significant differences were observed in community composition and functional profiles, which may reflect variations in sampling locations and study conditions. Our data set showed higher proportions of archaea (7.4% compared with 2.1%) and Actinomycetota (8.1% compared with <1%), consistent with microbial adaptations to the plateau environment characterized by low oxygen, low temperature, and intense UV radiation. Archaea, particularly methanogens, may enhance host energy efficiency under hypoxic conditions (42), while Actinomycetota are abundant in cold and oligotrophic habitats due to their production of bioactive metabolites that promote stress tolerance (43). Functionally, although the public data sets contained a greater number of ARGs, our MAGs exhibited resistance to a broader range of antibiotic classes (23 compared with 19).

To further explore the ARG profiles of Tibetan antelopes, we analyzed their gut-associated MAGs and identified 2,968 ARGs conferring resistance to 23 antibiotic classes. To place these findings in context, we compared the ARG composition of Tibetan antelopes with those of other plateau mammals and humans. Only 28 conserved ARG types were shared between humans and Tibetan antelopes, while seven were unique to the latter, indicating limited resistome overlap. This pattern likely reflects the sparse human presence on the Qinghai-Tibet Plateau, where extremely low population densities restrict anthropogenic antibiotic inputs (44).

While analyzing all ARGs in the gut microbiota of Tibetan antelopes, we found that resistance genes against elfamycin were the most prevalent. Elfamycins are a class of translation inhibitors that target elongation factor Tu (EF-Tu), thereby blocking bacterial protein synthesis (45). They have been mainly used in veterinary medicine and as potential growth promoters in livestock (46). Research on elfamycin remains insufficient, and due to its poor pharmacokinetic properties and solubility, it has mainly been used as a laboratory tool rather than in clinical applications. However, with the slowdown in the antibiotic development pipeline and the rapid emergence of resistance to approved antibiotics, elfamycins are now being reconsidered as potential therapeutic agents. Due to its limited clinical use, the likelihood of anthropogenic spread of elfamycin resistance is very low. This suggests that the presence of elfamycin resistance genes in Tibetan antelopes is more likely to reflect environmental or natural microbial reservoirs. For instance, previous studies have shown that elfamycin resistance is one of the more prevalent resistance types in wild rodents, further supporting this hypothesis (47). However, if transferred to human-associated bacteria, elfamycin resistance genes could compromise future therapeutic use of this antibiotic class and expand the environmental resistome (48). Continuous monitoring is therefore necessary to evaluate their potential mobility and cross-host dissemination risk.

The horizontal transfer of ARGs plays an important role in the global spread of antimicrobial resistance (49, 50), posing serious challenges to infection control and public health. To evaluate the potential for ARG dissemination within the gut microbiota of Tibetan antelopes, we examined the association between ARGs and MGEs. Correlation analysis revealed a significant positive relationship between MGE abundance and ARG diversity, suggesting that MGEs facilitate the maintenance and transmission of ARGs within the gut microbial community (51). Among the MGE types, insertion elements showed the strongest associations with ARGs, indicating that transposition may serve as a key mechanism driving ARG mobility in plateau microbiomes.

Overall, our findings provide new insights into the composition, mobility, and potential dissemination of ARGs in plateau wildlife microbiomes. Nevertheless, several limitations should be noted. First, the animal and human data sets were derived from different geographic regions, which restricts direct inference of resistance transmission pathways. Moreover, the limited availability of Tibetan human gut metagenomic data may underrepresent the true resistome landscape. In addition, while fecal samples from Tibetan antelopes were carefully collected in the field, we were unable to determine the sex, age, health status, or diet of the sampled individuals, which may influence gut microbiota composition and ARG profiles. These limitations highlight the need for future studies incorporating more comprehensive, spatially consistent sampling with detailed host metadata to clarify the dynamics of resistance dissemination between wildlife, livestock, and human populations.

### Conclusion

We conducted a comprehensive analysis of ARGs in Tibetan antelopes and untangled key findings. A high prevalence of resistance to elfamycin, glycopeptides, and aminoglycoside antibiotics was observed in the gut microbiota of Tibetan antelopes. These animals exhibited a unique combination of resistance genes, including seven distinct ARGs, most of which are associated with cross-resistance. The top ten most abundant ARGs were linked to virulence factors, suggesting enhanced pathogenicity. MGEs, especially insertion sequences, may play an important role in ARG transfer, showing a strong correlation between MGEs and ARG distribution. This association between ARGs and virulence factors indicates that Tibetan antelopes may face a higher risk of infection. Finally, we identified two viral sequences carrying ARGs that confer resistance to fusidane antibiotics and lincosamide antibiotics, respectively. Our findings highlight the complex interplay between resistance, virulence, and ecological factors. They underscore the need for further investigation into antibiotic resistance in wildlife and its implications for conservation and public health in plateau regions.

## Data Availability

The sequencing reads from each sequencing library have been deposited in CNGB and NCBI under the accession numbers: CNP0001390, PRJNA1257558, PRJEB53209, and PRJNA543906. All supplementary figures and tables are provided as additional files.

## SUPPLEMENTAL MATERIAL

Supplemental Material msystems.01443-25-s0001.docx Supplemental figures and captions for supplemental tables.

Supplemental Tables msystems.01443-25-s0002.xlsx Tables S1 to S13.

ASM does not own the copyrights to Supplemental Material that may be linked to, or accessed through, an article. The authors have granted ASM a non-exclusive, world-wide license to publish the Supplemental Material files. Please contact the corresponding author directly for reuse.
