# Supplementary material for: Comprehensive profiling of antibiotic resistance, virulence genes, and mobile genetic elements in the gut microbiome of Tibetan antelopes
Source: mSystems. 2025 Dec 23;11(2):e01443-25. doi: 10.1128/msystems.01443-25 (PMC12911420; doi:10.1128/msystems.01443-25)
Supplement: Supplemental Material — Supplemental figures and captions for supplemental tables. [file msystems.01443-25-s0001.docx]

**Supplementary Information**

**Comprehensive profiling of antibiotic resistance, virulence genes, and mobile genetic elements in the gut microbiome of Tibetan antelopes**

Jian Liu^a†^, Hong-Bo Ni^a†^, Ming-Yuan Yu^b†^, Si-Yuan Qin^b^, Hany M. Elsheikha^c^, Peng Peng^b^*, Li Guo^d^, Lin-Hong Xie^b^, Hong-Rui Liang^b^, Cong-Cong Lei^b^, Yu Xu^b^, Yan Tang^e^, Hai-Long Yu^a,f^, Ya Qin^a,f^, Jing Liu^g^, Hong-Chao Sun^h^, Xiao-Xuan Zhang^a^*, Bin Qiu^i^*

^a^ College of Veterinary Medicine, Qingdao Agricultural University, Qingdao, Shandong Province 266109, PR China.

^b^ Center of Prevention and Control Biological Disaster, State Forestry and Grassland Administration, Shenyang, Liaoning Province 110034, PR China.

^c^ Faculty of Medicine and Health Sciences, School of Veterinary Medicine and Science, University of Nottingham, Sutton Bonington Campus, Loughborough, United Kingdom.

^d^ Animal Science and Technology College, Jilin Agricultural Science and Technology University, Jilin, Jilin Province 132101, PR China.

^e^ College of Pharmacy, Guizhou University of Traditional Chinese Medicine, Guiyang, Guizhou Province 550025, PR China.

^f^ College of Veterinary Medicine, Jilin Agricultural University, Changchun, Jilin Province 130118, PR China.

^g^ College of Life Sciences, Changchun Sci-Tech University, Shuangyang, Jilin Province 130600, PR China.

^h^ Institute of Animal Husbandry and Veterinary Medicine, Zhejiang Academy of Agricultural Science, Hangzhou, Zhejiang Province 310021, PR China.

^i^ College of Chemistry and Pharmaceutical Sciences, Qingdao Agricultural University, Qingdao, Shandong Province 266109, PR China.

*Correspondence:

E-mail: pengpeng1782005@163.com (P. Peng); zhangxiaoxuan1988@126.com (X.X. Zhang); qiubin510105@163.com (B. Qiu)

^†^ These authors contributed equally to this work.

**Supplementary Fig. 1**: (A) MAG processing flow. (B) Completeness and contamination of the 13,600 MAGs. (C) GC content and genome size distribution of the 13,600 MAGs. (D) Sankey diagram illustrating species annotation of these MAGs.

**
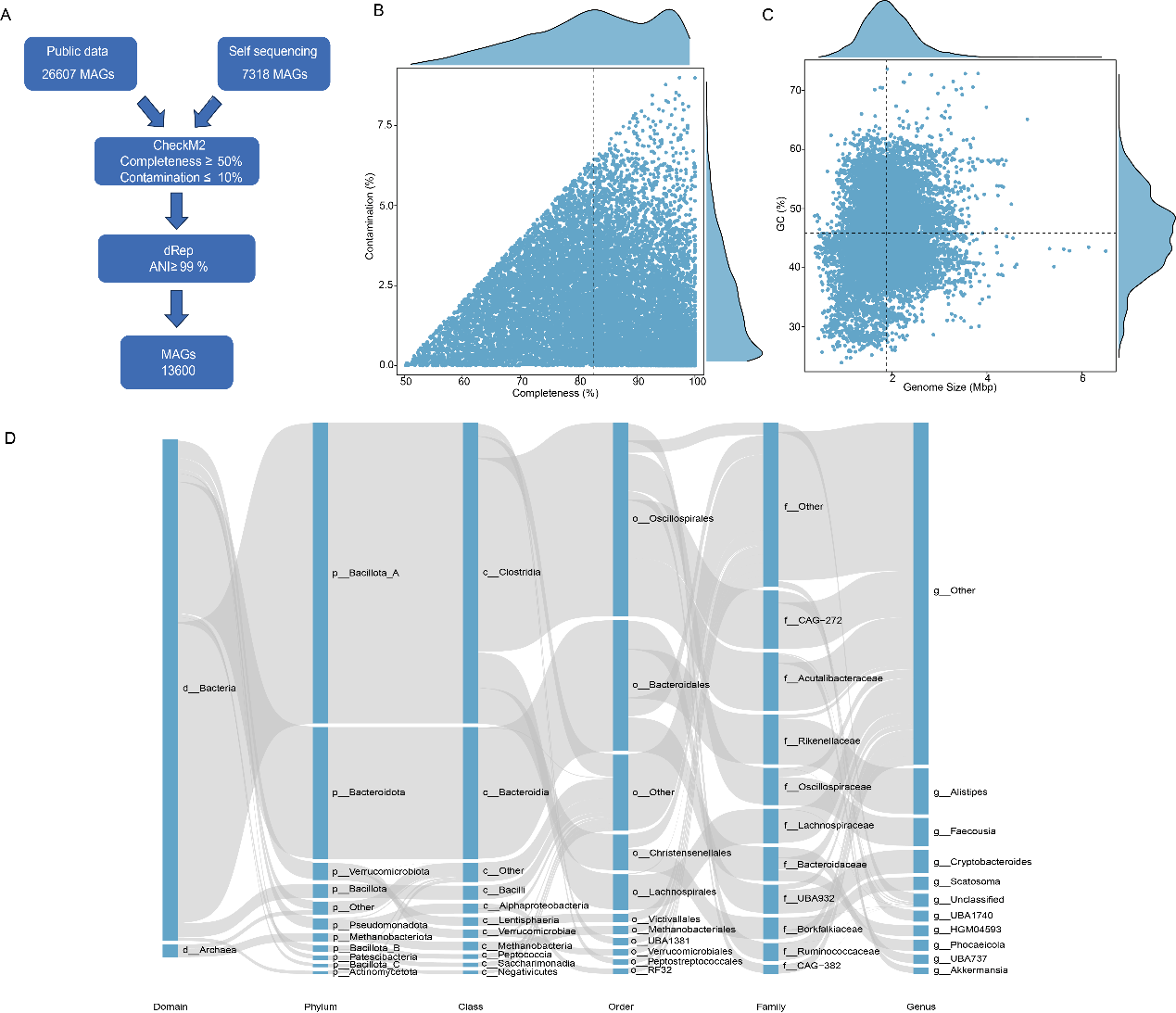
**

**Supplementary Fig. 2**: **Genomic Contribution Between VFGs and MGEs.** Circular genome maps are shown for three representative genomes. From the outermost to the innermost ring, annotations correspond to VFGs, MGEs, and ARGs, respectively(A)(B)(D)(E). The arrow diagram illustrates the genomic co-localization patterns of ARGs, MGEs, and VFGs(C)(F).

**
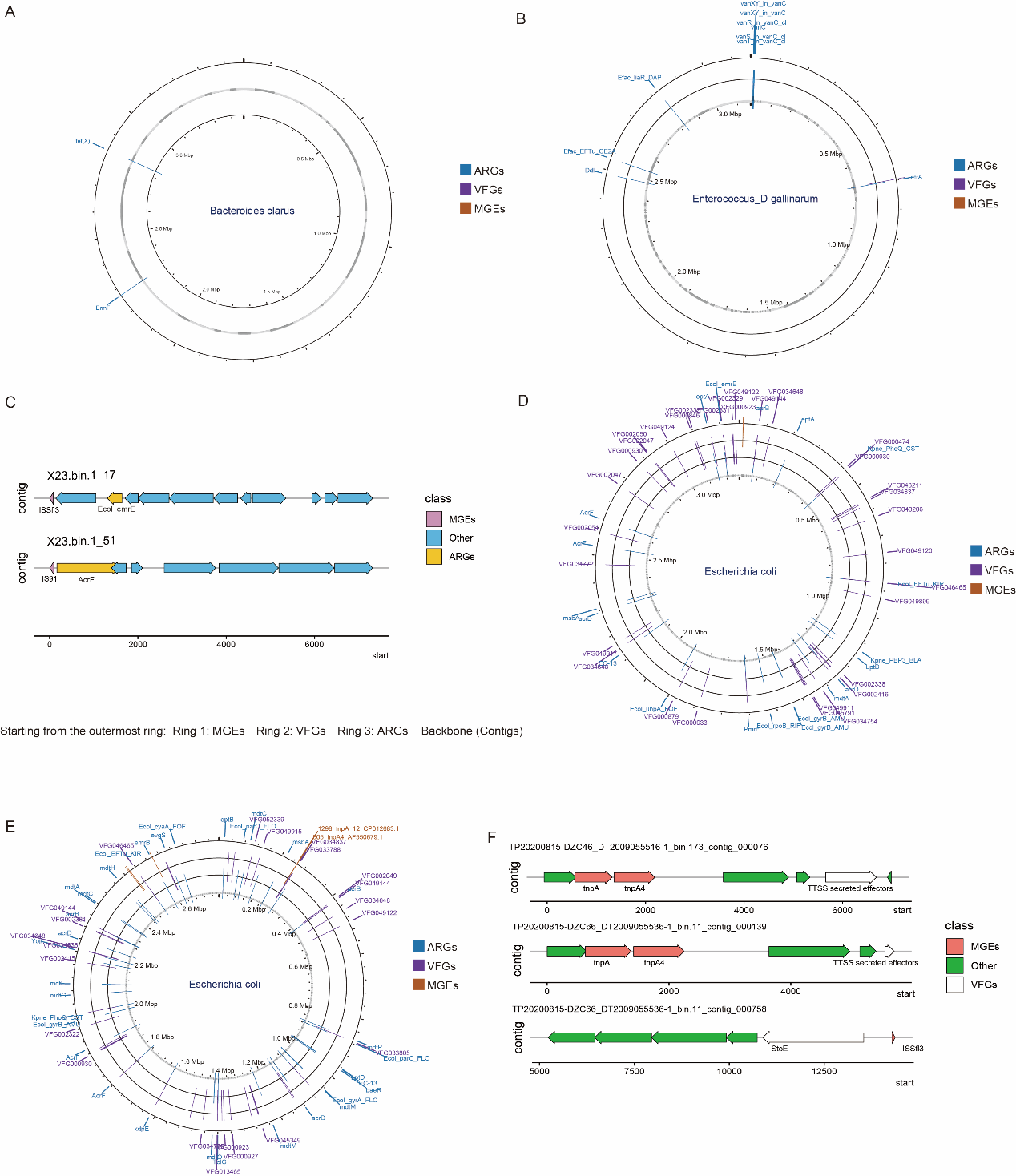
**

**Supplementary Table Legends:**

**Supplementary Table S1**: Project Collection Table.

**Supplementary Table S2**: Information of sample collections.

**Supplementary Table S3**: Species annotation information for Tibetan antelope MAGs.

**Supplementary Table S4:** Antibiotic resistance gene statistics for Tibetan antelopes.

**Supplementary Table S5:** ARGs blast result.

**Supplementary Table S6:** Comprehensive information on mobile genome elements in Tibetan antelopes.

**Supplementary Table S7:** Quantitative Profiling of MGEs and ARGs in MAGs from Tibetan Antelopes.

**Supplementary Table S8**: Comprehensive information on virulence genes in Tibetan antelopes.

**Supplementary Table S9**: Information on the collection of common ARGs against tigecycline, vancomycin, polymyxins, and β-lactams.

**Supplementary Table S10-S15:** Antibiotic resistance gene statistics for different species, including Tibetan ass, Tibetan cattle, Tibetan horse, Tibetan sheep, Yak, and Human, respectively.
